# Detection of articular perforations of the proximal humerus fracture using a mobile 3D image intensifier – a cadaver study

**DOI:** 10.1186/s12880-017-0201-0

**Published:** 2017-08-01

**Authors:** Jan Theopold, Kevin Weihs, Christine Feja, Bastian Marquaß, Christoph Josten, Pierre Hepp

**Affiliations:** 1 Department of Orthopedics, Trauma and Plastic Surgery, University of Leipzig, Liebigstrasse 20, 04103 Leipzig, Germany; 2 Institute of Anatomy, University of Leipzig, Liebigstrasse 13, 04103 Leipzig, Germany

**Keywords:** Proximal humerus fracture, Screw perforation, 3D imaging, Patient safety, Shoulder

## Abstract

**Background:**

The purpose of this study was to investigate the accuracy of perforation detection with multiplanar reconstructions using a mobile 3D image intensifier.

**Methods:**

In 12 paired human humeri, K-wires perforating the subchondral bone and placed just below the cartilage level were directed toward five specific regions in the humeral head. Image acquisition was initiated by a fluoroscopy scan. Within a range of 90°, 45° external rotation (ER) and 45° internal rotation (IR). The number and percentage of detected perforating screws were grouped and analyzed. Furthermore, the fluoroscopic images were converted into multiplanar CT-like reconstructions. Each K-wire perforation was characterized as “detected” or “not detected”.

**Results:**

In the series of fluoroscopy images in the standard neutral position at 30° internal rotation, and 30° external rotation, the perforations of all K-wires (*n* = 56) were detected. Twenty-nine (51.8%) of them were detected in one AP view, 22 (39.3%) in two AP views, and five (8.9%) in three AP views. All K-wire perforations (100%, *n* = 56) were detected in multiplanar reconstructions.

**Conclusion:**

In order to reveal all of the intraoperative and postoperative screw perforations in a “five screw configuration”, conventional AP images should be established in both the neutral positions (0°), at 30° internal rotation and 30° external rotation. Alternatively, the intraoperative 3D scan with multiplanar reconstructions enables a 100% rate of detection of the screw perforations.

## Background

Correct fracture classification, anatomical reduction, and stable fixation, along with avoidance of iatrogenic and material-related complications, may provide the basis for a good functional outcome following proximal humerus fracture surgery. In recent years, locking plates have been widely used for the treatment of proximal humerus fractures [1–4]. Nevertheless, high complication rates, comprising primary and secondary screw perforation, malreduction, malunion, nonunion, avascular necrosis, and infection, have been observed [5]. One area of particular concern involves the reportedly high rates of intraoperative humeral head screw perforation or screw cutout in the follow-up period [6–10]. This is likely the result of several factors, including diverging and converging locking screw vectors, the convex morphology of the humeral head, and poor bone quality limiting the tactile feedback of the drill bit, among other things. Iatrogenic articular screw penetration can lead to the destruction of the glenoid, which has been found to be unsatisfactorily treatable [7, 11]. The reduction is usually assessed intraoperatively while utilizing fluoroscopy in the anterior-posterior radiographic (AP) and Velpeau axillary views [12, 13]. The standard postoperative radiological control involves anteroposterior scapular, lateral scapular, and axillary radiographs.

The introduction of mobile 3D fluoroscopy has made intraoperative multiplanar imaging possible and it is used in navigated spinal surgery [14], pelvic operations [15], and for fractures of several extremeties [16–19]. Thus, the purpose of this study was to determine the AP views that are necessary to detect primary screw perforation of the humeral head under a controlled “in-vitro” setup. The secondary goal was to investigate the accuracy of perforation detection via multiplanar reconstructions using a mobile 3D image intensifier.

## Methods

### Specimen selection and preparation

Twelve paired human humeri were harvested from embalmed cadavers (two male and four female, mean age 76.8 years [range, 52–91 years]). All donors had given prior direct consent that their cadavers could be used for educational purposes or for research projects at the Institutes for Anatomy. Institutional review board approval was not required for this study. The specimens were dissected free of soft tissue, and biplanar radiographs were used to ascertain any bone abnormalities in the proximal humerus. Specimens with previous proximal humeral fractures, other underlying pathologic changes, or surgical intervention were excluded from the study.

Five 1.8 mm K-wires were guided into anterior, superior-anterior, inferior, superior-posterior, and posterior positions using a locking plate with a targeting device (Winsta PH, Axomed, Freiburg, Germany), which ensured a reproducible placement of the K-wires. The wire placement was performed by a single surgeon experienced in shoulder surgery (JT).

**Fig. 1:**
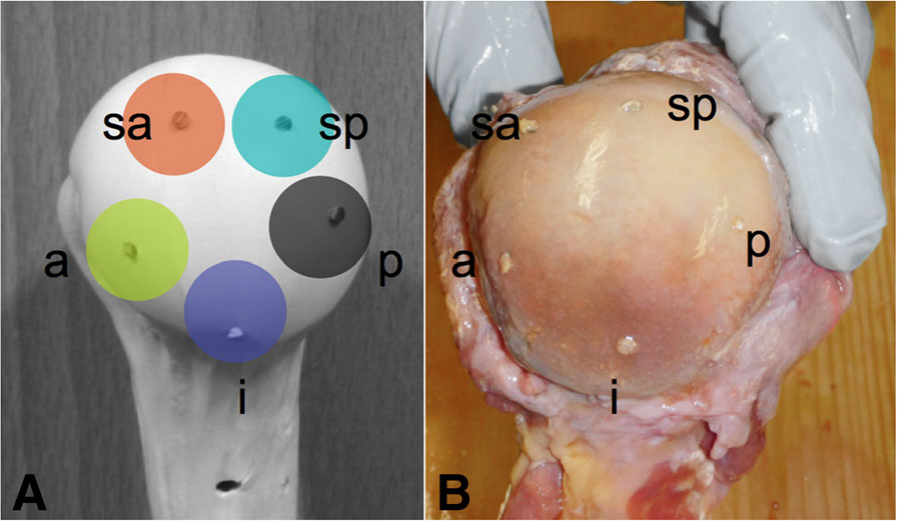
Regions of articular K-wire perforation; **a** schematic view on a synbone and **b** subchondral placement of K-wires in a specimen in the following positions: a = anterior, sa = superior-anterior, i = inferior, sp = superior-posterior, and p = posterior

Each proximal humerus was positioned horizontally to ensure the maximum projection of the greater humeral tuberosity on a two-dimensional AP view. The perforations were verified by confirming them visually (Fig. 1).

### Fluoroscopic imaging and detection of perforation

Image acquisition was initiated by performing a fluoroscopy scan with the mobile Ziehm Vision FD Vario 3D^©^ (Ziehm Imaging GmbH, Nurnberg, Germany). The motorized fluoroscope features a variable isocentric C-arm design and collects 110 fluoroscopic images during a 135° arc of rotation around an anatomic region of interest. Its isocenter is held in place to allow for the movements of the C-arm cantilever. This readjustment is automated.

**Fig. 2:**
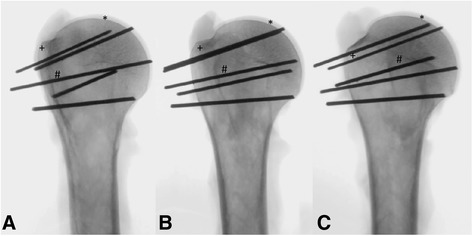
Detection of K-wire perforations in different positions of the humeral head. **a** 30° external rotation (ER), **b** 0°, **c** 30° internal rotation (IR); *cartilage, +greater tuberosity, #lesser tuberosity

Each fluoroscopic image was analyzed (MagicWeb VA60C_0212, Visage Imaging GmbH Berlin, Germany) by three of the authors (JT, PH, KW) and K-wire perforation was documented for all five positions (Fig. 2).

As each image corresponds to a 1.23° step and the perforation was visible in consecutive images, the “angle of visible perforation” (AVP) for each K-wire was calculated by multiplying the number of images with visible perforation (NVP) by 1.23: AVP = NVP*1.23.

The neutral position was set to 0° which matched the classical AP view of the shoulder joint and corresponded to fluoroscopy image number 73. An external rotation (ER) of 45° corresponded to image number 37, while an internal rotation (IR) of 45° corresponded to image number 110. The analysis of K-wire perforation was performed within a 90° range (45° IR to 45° ER; image 37–110; *n* = 73 images). The number and percentage of detected perforating screws were grouped and analyzed for each of the two series of AP views: 30° IR – 0°–30° ER and 45° IR – 0°–45° ER.

**Fig. 3:**
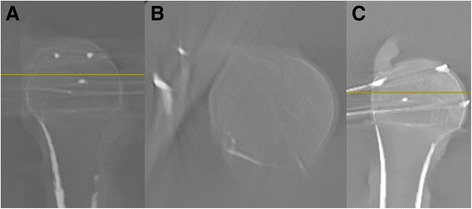
Screenshots after the 3D scan of a proximal humerus; **a** sagittal plane, **b** axial plane, **c** coronal plane

All 110 of the fluoroscopic images were then converted into multiplanar CT-like reconstructions using the Ziehm software version 5.63 (Ziehm Imaging GmbH, Nurnberg, Germany). On the workstation, each wire was identified in coronal, axial, and sagittal views (Fig. 3). The correct placement of each K-wire was verified and each K-wire perforation was characterized as “detected” or “not detected”. K-wires that did not match the aforementioned inclusion criteria were excluded from further analysis.

The fluoroscopy images, the multiplanar reconstructions and the specimen of the non-detected perforations were subsequently reevaluated. This revealed a secondary displacement of four wires. One K-wire did not perforate the subchondral bone. Three further K-wire perforations were visible in all 110 images. The analysis of the specimen revealed a secondary K-wire dislocation. For all remaining k-wires the initial placement was confirmed.

### Statistics

All data were collected in a computerized database. The data was analyzed by means of descriptive statistics (SPSS, version 20, Chicago, IL, USA). A chi square test was used to test differences in the number of visible perforations in the different positions. Cronbach’s Alpha statistic was used to evaluate inter-observer error in image analysis. The significance level was set to *p* < 0.05.

## Results

### Detection of perforation

**Fig. 4:**
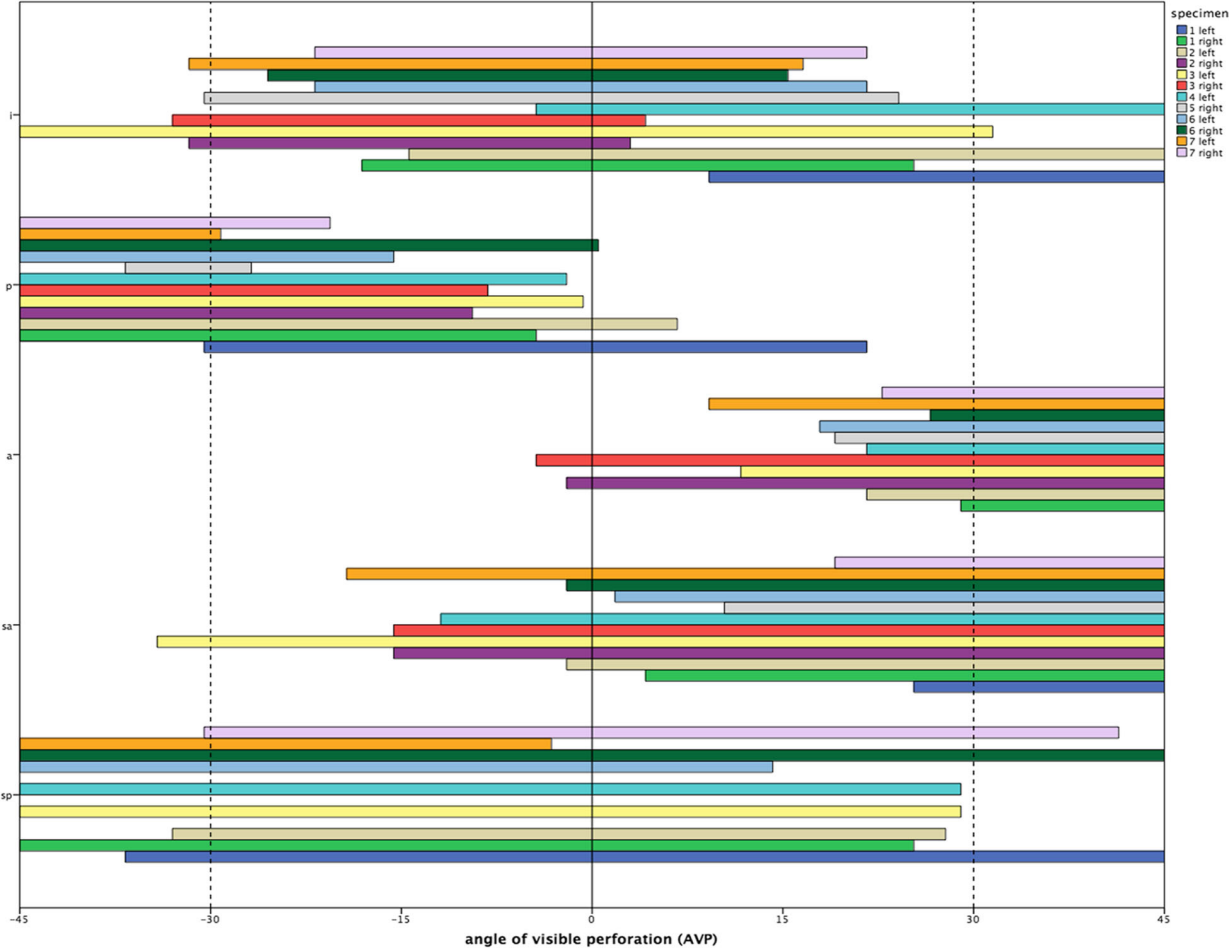
Angle of visible perforation (AVP) (−45°/0°/45°) for each single K-wire; i = inferior, p = posterior, a = anterior, sa = superior anterior, and sp = superior-posterior

All 56 K-wire perforations (100%) were detected on the fluoroscopy images of the 12 specimens (Table 1). A high inter-observer reliability of 0.93 (Cronbach’s Alpha) was found. The perforating K-wires of the 12 specimens were detected at an angle of visible perforation (AVP) of mean 29.3° (between 45° IR and 15.7° IR) in the anterior position, 48.3° (between 45° IR and 3.3° ER) in the superior-anterior position, 47.3° (between 25° IR and 22.4° ER) in the inferior position, 69.3° (between 28.2° IR and 41.1° ER) in the superior-posterior position, and 35.8° (between 7.3° ER and 43.1° ER) in the posterior position (Fig. 4).

**Table 1:** Number (%) of detected K-wire perforations in different AP views

Location of perforation	Arm position				
	45° ER	30° ER	0°	30° IR	45° IR
Superior-posterior	6/9	9/9	8/9	3/9	2/9
Superior-anterior		1/12	7/12	12/12	12/12
Anterior			2/11	11/11	11/11
Posterior	10/12	12/12	3/12		
Inferior	1/12	5/12	11/12	4/12	3/12
Total (*n* = 56)	17 (30.4%)	27 (48.2%)*	31 (55.4%)	30 (53.6%)	28 (50%)

*significantly more perforations were detected in 30° external rotation (ER) compared to 45° ER (*p* = 0.041)

On the series of fluoroscopy images in the standard neutral position at 30° internal rotation, and at 30° external rotation, perforations of all K-wires (*n* = 56) were detected. Twenty-nine (51.8%) of them were detected in one AP view, 22 (39.3%) in two AP views, and five (8.9%) in three AP views.

In the “45° IR – 0°–45° ER” series, one perforation was not detected (1.8%, one posterior K-wire), while 35 (62.5%) of them were detected in one AP view, 19 (33.9%) in two AP views, and one (1.8%) in three AP views. The one perforation that was not detected in this series was only visible at an angle of 26.8° ER to 36.7° ER. Therefore, the view in 45° ER did not detect the perforating screw.

Significantly more perforations were detected in 30° ER compared to 45° ER (*p* = 0.041). There were no significant differences between the other AP views (*p* > 0,05).

All K-wire perforations (100%, *n* = 56) were detected in the multiplanar reconstructions and the coronal reconstruction offered the best visibility (Fig. 3).

## Discussion

The principal findings of this study show that a combination of three AP views – neutral, 30° internal rotation, and 30° external rotation – permit the identification of 100% of articular perforations in an in-vitro setup. Additionally, coronal reconstruction of a 3D fluoroscopic scan provided a 100% rate of detection of the perforating K-wires.

The use of locking plates in the surgical treatment of proximal humerus fractures is associated with an unexpectedly high rate of screw cutouts and revision surgery [9]. Biomechanical studies have emphasized the value of anchoring screws in the subchondral bone of the humeral head to improve implant stability [20, 21]. However, the spherical shape of the proximal humerus and the limited tactile sensation of its soft cancellous bone make it difficult to determine an accurate screw length, and reported rates of intraoperative screw penetration are high. Iatrogenic screw penetration, even if recognized and corrected before leaving the operating room, may lead to late failure [11].

The protocol for using locking plates and the attention placed on the technical aspects of applying them have been emphasized in the past [11, 22]. Nevertheless, only a few studies have investigated the potential for optimizing the recognition of early or late stage screw perforation [23, 24].

Complications following proximal humerus fracture surgery may likely be a result of inadequate use of intraoperative radiographs or fluoroscopy [25]. Accordingly, the use of routine intraoperative fluoroscopy to confirm hardware placement and a stable anatomical reduction are recommended. The anteroposterior view is a key component of a basic shoulder series. Often, two AP projections are obtained, namely one with the arm in an external rotation and one in an internal rotation [26]. Nevertheless, the algorithm for adequate intraoperative imaging remains inconsistent. Bengard and Gardner suggested placing the arm at 20° to 30° flexion and 80° to 90° internal rotation to aid in visualizing the difficult-to-assess posterosuperior region of the humeral head [11]. However, in contrast to our study they did not provide experimental data to corroborate their suggestion. Spross et al. identified a combination of four projections to account for all cut outs and to establish the correct screw position [23]. In a cadaver study, they determined that the axial view with 30° abduction was the best radiographic projection (76% sensitivity), and that a combination of four views (APIR/AP0°/APER/ax30°) had a sensitivity of 100%. They too examined a combination of the external rotation, neutral position and internal rotation (sensitivity 96%), though the degree of internal rotation (sling position) appeared variable. With intraoperative 3D fluoroscopy and multiplanar reconstruction standardized imaging with CT-like quality can be obtained. At the same time the findings of our study suggest that the investigated procedure holds the potential to detect 100% of primary screw perforations as one of the most common intraoperative complications. Recently, Lowe et al. described the use of a combination of nine fluoroscopic images to identify eight of nine intra-articular screws with a sensitivity of 100% [24]. Their recommendation to use nine C-arm views to evaluate screw placement may be realistic under in-vitro conditions with standardized placement of the screws.

Moreover, all screws may be scrutinized under fluoroscopy in varying degrees of internal and external rotation in order to verify that there is no need for intra-articular hardware [12].

We suggest the use of intraoperative 3D scans to detect 100% of screw perforations independent of screw placement. The advantage of the intraoperative fluoroscopic 3D scan compared to conventional live fluoroscopy is the defined number of images together with multiplanar reconstruction. Moreover, the operating personnel can leave the operating room which reduces the radiation exposure. For the analyzed five screw configuration at least four determined images (30° IR, 30° ER, AP, axial) are needed to examine all screws properly. Finding the right plane involves several control images especially as an exact axial plane is not reproducible with certainty. Altogether this would lead again to a higher amaount of radiation exposure at least for the personnel in the operating room.

The important role of intraoperative multiplanar imaging after osteosynthesis is supported by the high rate of immediate corrections for 11–39% of other regions of the body [16, 18, 19, 27]. Hence, the additional medical benefit seems undisputed [28].

In a feasibility study of the intraoperative use of a mobile 3D C-arm with multiplanar imaging for operating on acute proximal humerus fractures in 20 patients, screw replacements due to perforation or subchondral positioning were performed in 25% of cases [29]. Overall, the complication and revision rates due to technical errors after locking plate osteosynthesis [30, 31] may be drastically reduced if flaws were discovered intraoperatively. The question of whether or not the making of intraoperative corrections using 3D scans leads to superior immediate and long-term functional results has not yet been sufficiently investigated for other joints [32].

Our study has the same inherent weakness of many cadaveric studies. However, the mean age of the cadavers used in our study was 76.8 years, corresponding to the typical age of patients undergoing surgery after proximal humerus fractures. In addition, the results of our study are only valid for the tested plate design with the five screw configuration and are not generalizable. Other proximal humeral plate systems and screw configurations would need separate testing to determine the necessary X-ray views for the detection of all perforations. Whereas most implant designs have at least eight options for screw placement, a screw configuration with five screws has been chosen for the present study. This is in accordance with Erhardt et al. [33] who suggested that at least five screws in the humeral head fragment are necessary for the stabilization of proximal humeral fractures. The screw configuration that was used was defined by the angle stable locking plate and the necessary targeting device. Nevertheless, additional X-ray images may be necessary for other screw configurations [23, 24] and plate designs. Despite the positive aspect of our findings, namely the successful identification of all screw perforations with three AP views, additional X-ray images are often required to properly assess reduction and fixation. Therefore, the intraoperative 3D scan with multiplanar reconstruction is optimal in that it detects all perforations, independent of the screw configuration, and provides critical information regarding reduction and fixation. Finally, the radiation exposure of the intraoperative 3D scan may be a major concern, though in comparison to computed tomography, the radiation dose is significantly reduced. However, to our knowledge comparative data are not available in the literature.

## Conclusion

In order to reveal all of the intraoperative and postoperative screw perforations in a “five screw configuration”, conventional AP images should be established in both the neutral positions (0°), at 30° internal rotation and 30° external rotation. Alternatively, the intraoperative 3D scan with multiplanar reconstructions enables a 100% rate of detection of the screw perforations.

## Abbreviations

3D: Three-dimensional
AP: Anterior-posterior
AVP: Angle of visible perforation
CT: Computed tomography
ER: External rotation
IR: Internal rotation
K-Wire: Kirschner wire
NVP: Number of images with visible perforation
